# The prognostic value of miR-34 family in ovarian cancer: a systematic review and meta-analysis

**DOI:** 10.3389/fonc.2025.1499163

**Published:** 2025-03-17

**Authors:** Xiulan Luo, Xiaolu Li, Chaolin Chen, Jing Yang, Hong Zheng

**Affiliations:** Department of Pathology, Affiliated Hospital of Zunyi Medical University, Zunyi, Guizhou, China

**Keywords:** meta-analysis, microRNA, miR-34s, ovarian cancer, prognosis

## Abstract

**Background:**

The aim of this study was to evaluate the association between miR-34 family expression and overall survival (OS) and progression-free survival (PFS) in women with ovarian cancer.

**Methods:**

Literature searches were conducted using databases such as PubMed, Cochrane Library, EMBASE, Web of Science, Wanfang, and CNKI to identify studies reporting pooled hazard ratios (HRs) and 95% confidence intervals (CIs) examining the relationship between miR-34 family expression and overall survival (OS) or progression-free survival (PFS) in female patients with ovarian cancer. All potentially relevant studies were assessed and then pooled.

**Results:**

There were a total of seven literatures included in this systematic review and meta-analysis, which included 672 women. There was a significant improvement in survival for women with ovarian cancer when miR-34s expression was higher (OS, HR = 0.70, 95% CI:0.57–0.86; PFS, HR = 0.48, 95% CI:0.31–0.75). A subgroup analysis of miR-34 family members showed that differences between groups greatly affected PFS (HR = 0.50, 95% CI: 0.40–0.63).

**Conclusion:**

Based on the results of this review, it appears that ovarian cancer women with high expression of miR-34s may have a better chance of surviving.

Systematic Review Registration: PROSPERO (CRD42024499203).

**Systematic review registration:**

https://www.crd.york.ac.uk/PROSPERO/, identifier CRD42024499203.

## 1 Introduction

Among female reproductive system tumors, ovarian cancer is the most common, and it is one of the most aggressive (1). Ovarian cancer is usually diagnosed at an advanced stage with a poor prognosis due to the absence of clinical symptoms in the early stages and the high failure rate of chemotherapy (2). It is estimated that in 2020, there will be around 3,13,959 new cases of ovarian cancer globally, with an incidence of 6.6 and a mortality rate of 4.4, according to the data survey (3). In spite of improvements in survival rates for women with ovarian cancer in the past few decades, the 5-year survival rate for women with stage I epithelial ovarian cancer remains below 30%. It is well known that the miR-34 family (miR-34s) is essential for the pathogenesis of epithelial ovarian cancer, and its inactivation might have significant implications for its progression (2). Therefore, in order to improve the prognosis of ovarian cancer patients, biomarkers relating to prognosis must be identified and new therapeutic targets explored.

There are approximately 22 nucleotides in the length of microRNA (miRNA; miR) that are endogenous non-coding RNA. The miRNA negatively regulates gene expression at the post-transcriptional and translational levels, and plays a significant role in cancer progression (4). miR-34s is a family of three miRNAs that is encoded by two different genes in the human body, miR-34a, miR-34b, and miR-34c. There is an individual transcript for miR-34a on chromosome 1p36.22; however, miR-34b and miR-34c share the same chromosome 11q23.1 for transcription and expression (5). Several processes in tumor development are regulated by the tumor suppressor miR-34s, which is low expressed in most tumors, including proliferation, migration, invasion, metabolism, apoptosis, and cancer stem-cell formation, cell cycle progression, epithelial mesenchymal transformation, and so on, the result is an inhibition of tumor growth and metastasis (2, 6). Direct transcriptional targets of p53 have been shown to be miR-34a and miR-34b/c (7). According to Hannah Welponer et al. (8)show that The levels of miR-34a/b/c in ovarian cancer were found to be significantly decreased compared to control tissues (*p*=0.002, *p*<0.001, *p*<0.001, respectively). Furthermore, the expression of each member of the miR-34 family was determined to have independent prognostic significance in relation to progression-free survival (PFS) (miR-34a: HR=0.6, *p*=0.033; miR-34b: HR=0.2, *p*=0.001; miR-34c: HR=0.3, *p*=0.002, respectively). It was determined that miR-34b (HR=0.4, *p*=0.016) and miR-34c (HR=0.6, *p*=0.049) have a prognostic value for overall survival (OS). Research conducted by Jia et al. (9) found no correlation between the presence of miR-34a and overall survival (HR=1.407, *p*=0.929) or progression-free survival (HR=0.855, *p*=0.727) in individuals with epithelial ovarian cancer. However, a study by Song et al. (10) found significant effects of tumor size, FIGO stage, histological type, and miR-34c expression on overall survival in ovarian cancer patients. An analysis of multivariate data found a significant association between miR-34c expression and shorter OS time (*p*=0.038). Consequently, it is necessary to evaluate miR-34s expression and ovarian cancer survival through meta-analysis in order to determine the diagnosis and prognosis assessment of the miR-34s.

In this study, in order to obtain more accurate information about the prognosis of ovarian cancer, miR-34s expression needs to be further studied. Reviewing relevant literature and using systematic reviews and meta-analyses to assess miR-34s prognostic value in ovarian cancer was the goal of this study.

## 2 Materials and methods

### 2.1 literature search strategy

We searched six electronic databases was conducted for studies published from 2007 until April 2023. and their creation (PubMed, Cochrane Library, EMBASE, Web of Science, Wanfang, and CNKI) for relevant literature. Based on the PICOS tool, a search strategy was formulated: (P) Population: Ovarian cancer patients; (I) Intervention: high expression of miR-34s; (C) Comparator: low expression of miR-34s; (O) Outcomes: OS or PFS; (S) Study Design: retrospective or prospective studies. An overview of the search strategy can be found in **Table 1** and **Supplementary Materials** (The example used here is PubMed).

**Table 1:** PubMed database strategy to conduct searches.

#1	(Ovarian Neoplasms [MeSH Terms]) OR (Carcinoma, Ovarian Epithelial [MeSH Terms])
#2	(Neoplasm, Ovarian[Title/Abstract])) OR (Ovarian Neoplasm[Title/Abstract])) OR (Neoplasm, Ovary[Title/Abstract])) OR (Neoplasms, Ovary[Title/Abstract])) OR (Ovary Neoplasm[Title/Abstract])) OR (Neoplasms, Ovarian[Title/Abstract])) OR (Ovary Cancer[Title/Abstract])) OR (Cancer, Ovary[Title/Abstract])) OR (Cancers, Ovary[Title/Abstract])) OR (Ovary Cancers[Title/Abstract])) OR (Ovarian Cancer[Title/Abstract])) OR (Cancer, Ovarian[Title/Abstract])) OR (Cancers, Ovarian[Title/Abstract])) OR (Ovarian Cancers[Title/Abstract])) OR (Cancer of Ovary[Title/Abstract])) OR (Cancer of the Ovary[Title/Abstract])) OR (Epithelial Carcinoma, Ovarian[Title/Abstract])) OR (Ovarian Epithelial Carcinomas[Title/Abstract])) OR (Epithelial Ovarian Cancer[Title/Abstract])) OR (Ovarian Epithelial Cancer[Title/Abstract])) OR (Cancer, Ovarian Epithelial[Title/Abstract])) OR (Epithelial Cancer, Ovarian[Title/Abstract])) OR (Ovarian Epithelial Cancers[Title/Abstract])) OR (Ovarian Cancer, Epithelial[Title/Abstract])) OR (Cancer, Epithelial Ovarian[Title/Abstract])) OR (Epithelial Ovarian Cancers[Title/Abstract])) OR (Ovarian Epithelial Carcinoma[Title/Abstract])) OR (Epithelial Ovarian Carcinoma[Title/Abstract])) OR (Carcinoma, Epithelial Ovarian[Title/Abstract])) OR (Epithelial Ovarian Carcinomas[Title/Abstract])) OR (Ovarian Carcinoma, Epithelial[Title/Abstract])) OR (Ovary Neoplasms[Title/Abstract])
#3	#1 OR #2
#4	MIRN34 microRNA, human [MeSH Terms]
#5	(hsa-mir-34 microRNA[Title/Abstract])) OR (miR-34, human[Title/Abstract])) OR (MIRN34B microRNA, human[Title/Abstract])) OR (hsa-mir-34b microRNA[Title/Abstract])) OR (microRNA-34b, human[Title/Abstract])) OR (MIRN34C microRNA, human[Title/Abstract])) OR (hsa-mir-34c microRNA[Title/Abstract])) OR (miR-34c, human[Title/Abstract])) OR (microRNA-34c, human[Title/Abstract])) OR (miR-34b-3, human[Title/Abstract])) OR (miR-34c-5p, human[Title/Abstract])) OR (Pri-miR-34b-c, human[Title/Abstract])) OR (MIRN34A microRNA, human[Title/Abstract])) OR (miR-34a, human[Title/Abstract])) OR (microRNA-34a, human[Title/Abstract])) OR (hsa-mir-34a microRNA[Title/Abstract])) OR (microRNA 34a, human[Title/Abstract])
#6	#4 OR #5
#7	#3 AND #6

### 2.2 Inclusion criteria

The results of studies were included if they evaluated the following: (1) OS/PFS of ovarian cancer patients and miR-34s expression; (2) A sufficient amount of information was provided to calculate hazard ratios (HRs) and 95% confidence intervals (CIs); and (3) Retrospective or prospective studies.

### 2.3 Exclusion criteria

(1) Data that is incomplete or unreported in studies; (2) Study results from non-observational studies[including reviews, cell experiments, animal experiments, correspondence, protocols, conference abstracts, or case reports.

### 2.4 Study selection

Utilizing the literature management software NoteExpress 3.8, we screened and excluded the literature. Research was conducted by two researchers to identify duplication, review papers, conference papers, protocols, and correspondence in the literature. It was then determined which literature should be included and which should be excluded by two researchers after reading the abstracts. Finally, literature not included in the study was read in full by both researchers and was identified for inclusion. This process involved both researchers screening the literature independently, then comparing the remaining literature. Those who remained the same were included, and those who differed were brought in to discuss and resolve the differences.

### 2.5 Data extraction

Data were extracted independently by two reviewers. Data were collected using nine standardized and preselected data extraction forms under the following headings for inclusion in the study: (1) author, (2) date of publication, (3) nation,(4) Study design,(5) miRNA, (6)miRNA cut-off value,(7) disease type, (8) mean age, (9) sample size, (10) assay, and (11) outcome. A 95% CI and *p* value are provided for the HRs of miR-34s expression for OS and PFS. In the absence of direct reporting of HR and 95% CI, we calculated them based on the number of reported patients and events observed in each group. The HRs were estimated from graphical survival plots if only Kaplan-Meier curves were available (11). In cases where univariate and multivariate analyses were reported simultaneously, only the multivariate analysis was analyzed. Due to the fact that multivariate analysis accounted for confounding factors, the results were more valuable. It was resolved through discussion among the first three authors that differences over data extraction could be resolved.

### 2.6 Individual studies may be biased

To evaluate included studies, independent researchers used the Newcastle Ottawa scale (NOS), a method for assessing the quality of observational studies and non-randomized trials. Study groups are compared based on three factors: participant selection, comparability of results, and outcome assessment. In total, nine stars were awarded, one for each item, with the exception of the item “Comparability of cohorts based on design or analysis,” which was awarded two stars. Studies with quality scores less than 6 are considered low-quality studies. A study with a quality score of at least 6 is considered to be included in the meta-analysis. This meta-analysis follows the PRISMA guidelines. Under registration number CRD42024499203, it is registered with PROSPERO (International Register of Prospective Systems Evaluation).

### 2.7 Data analysis

A comprehensive statistical analysis was conducted using RevMan 5.4 software. OS and PFS were defined as the primary outcome indices. The hazard ratio (HR) and its corresponding 95% confidence interval (CI) were utilized as effect indices, calculated using the inverse variance (IV) method.

To assess the heterogeneity among included studies, we applied Cochran’s Q test and calculated the I² statistic (12). If the I² value was less than or equal to 50% and the p-value was greater than or equal to 0.1, it indicated no significant heterogeneity among studies, and a fixed effects model was employed for the meta-analysis. Conversely, if the I² value exceeded 50% and the *p*-value was less than 0.1, indicating significant heterogeneity, we opted for a random effects model. In the event of high heterogeneity, we conducted subgroup analyses stratified by individual members of the miR-34 family (miR-34a, miR-34b, and miR-34c) to explore potential sources of variability. Additionally, we performed sensitivity analyses to further investigate the origin of the observed heterogeneity. To address the possibility of publication bias, we generated funnel plots for visual assessment.

## 3 Results

### 3.1 Study and identification and selection

**Figure 1:**
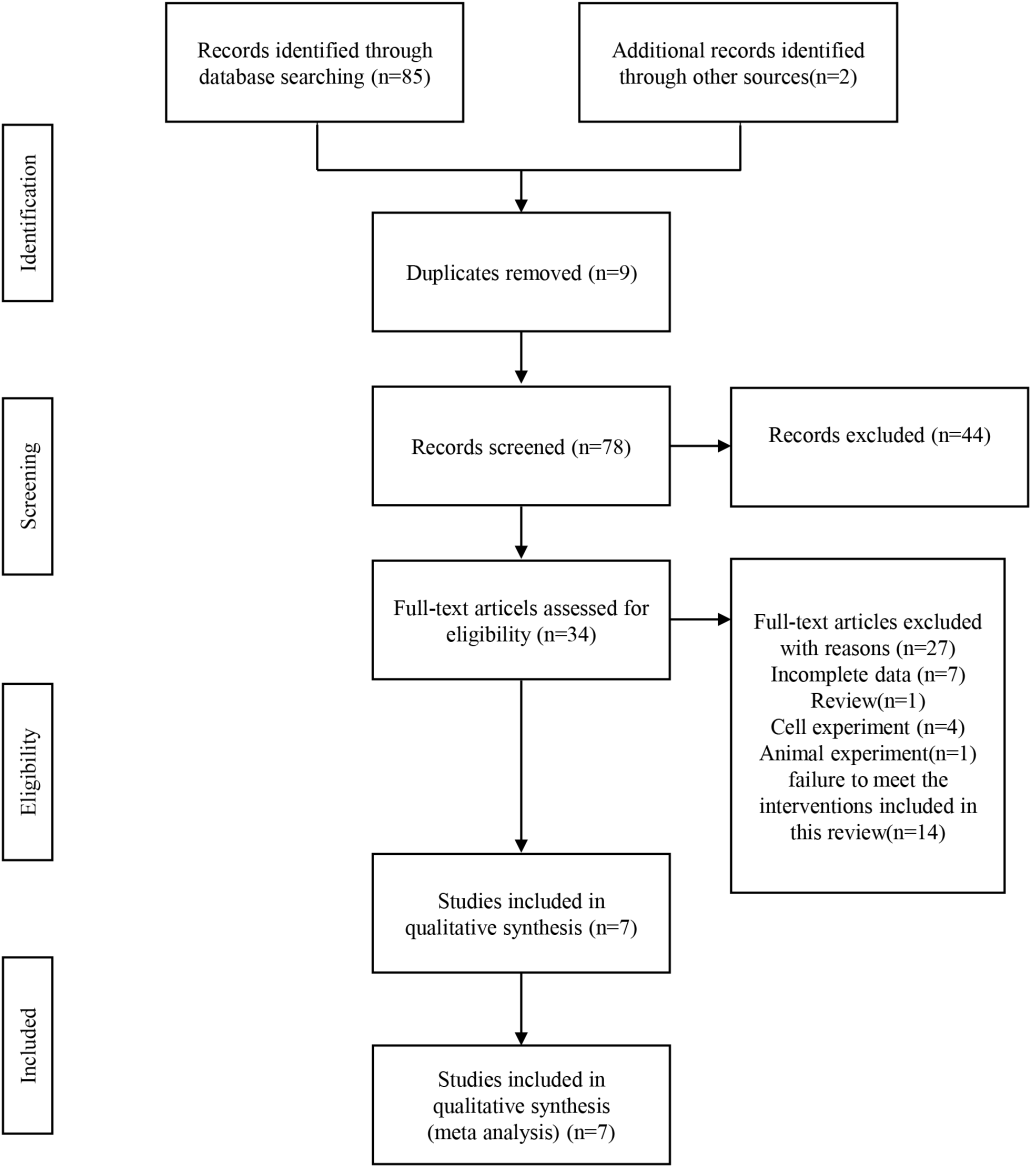
A flow chart for the systematic review process according to PRISMA.

An additional two literatures were manually searched in addition to 85 literatures retrieved from six electronic databases. Following the elimination of duplicates, to review the remaining 78 literatures, we read their titles and abstracts, and 34 papers were then excluded again. In total, 34 literatures were read in full, but 27 literatures were excluded (due to incomplete data, review, cell experiment, animal experiment, and interventions included in the review were not met). A total of 7 literatures remain for inclusion in this study. During the period 2009 to 2020, there were 7 literatures published including 672 ovarian cancer patients. Different ethnic groups were involved: 3 from China, 3 from Austria and 1 from Canada. miRs were mainly expressed in tissue samples. The detection method was qRT-PCR. In all 7 studies, 6 studies had survival indicators of OS and 5 studies had survival indicators of PFS. As shown in **Figure 1**, the included studies have a variety of characteristics.

### 3.2 An assessment of the quality of the included studies

7 literatures were eligible for inclusion, of which one was of low quality and eight were of medium quality, mainly because none of the papers described the non-response rate (**Table 2**).

**Table 2:** The meta-analysis included the following characteristics.

Author	Year	Nation	Study design	miRNA	miRNA cut-off value	Disease	Age(mean ± SD)	Sample size	Assay	Outcome	NOS score
Lee (13)	2009	Canada	Retro	miR-34c	Median	High grade serous carcinomas	NR	33	qRT-PCR	PFS	7
Reimer (14)	2011	Austria	Retro	miR-34a	Median	Epithelial ovarian cancer	NR	130	qRT-PCR	OS, PFS	8
Liu (9)	2015	China	Retro	miR-34a	Median	Epithelial ovarian cancer	57.48 ± 11.77	44	qRT-PCR	OS, PFS	8
Schmid (15)	2016	Austria	Retro	miR-34a	Median	Epithelial ovarian cancer	62.3	133	qRT-PCR	OS, PFS	8
Dong (16)	2016	China	Retro	miR-34a	Median	Serous ovarian cancer	NR	50	qRT-PCR	OS	6
Xiao (10)	2018	China	Pro	miR-34c	Median	Ovarian cancer	NR	54	qRT-PCR	OS	8
Welponer (8)	2020	Austria	Retro	miR-34a	Median	Epithelial ovarian cancer	61	228	qRT-PCR	OS, PFS	8
Welponer (8)	2020	Austria	Retro	miR-34b	Median	Epithelial ovarian cancer	61	228	qRT-PCR	OS, PFS	8
Welponer (8)	2020	Austria	Retro	miR-34c	Median	Epithelial ovarian cancer	61	228	qRT-PCR	OS, PFS	8

*Pro* prospective, *Retro* retrospective, *NR* not reported, *OS* overall survival, *PFS* progress-free survival, *NOS* Newcastle Ottawa scale.

### 3.3 Meta-analysis for miR-34s

#### 3.1.1 OS

**Figure 2:**
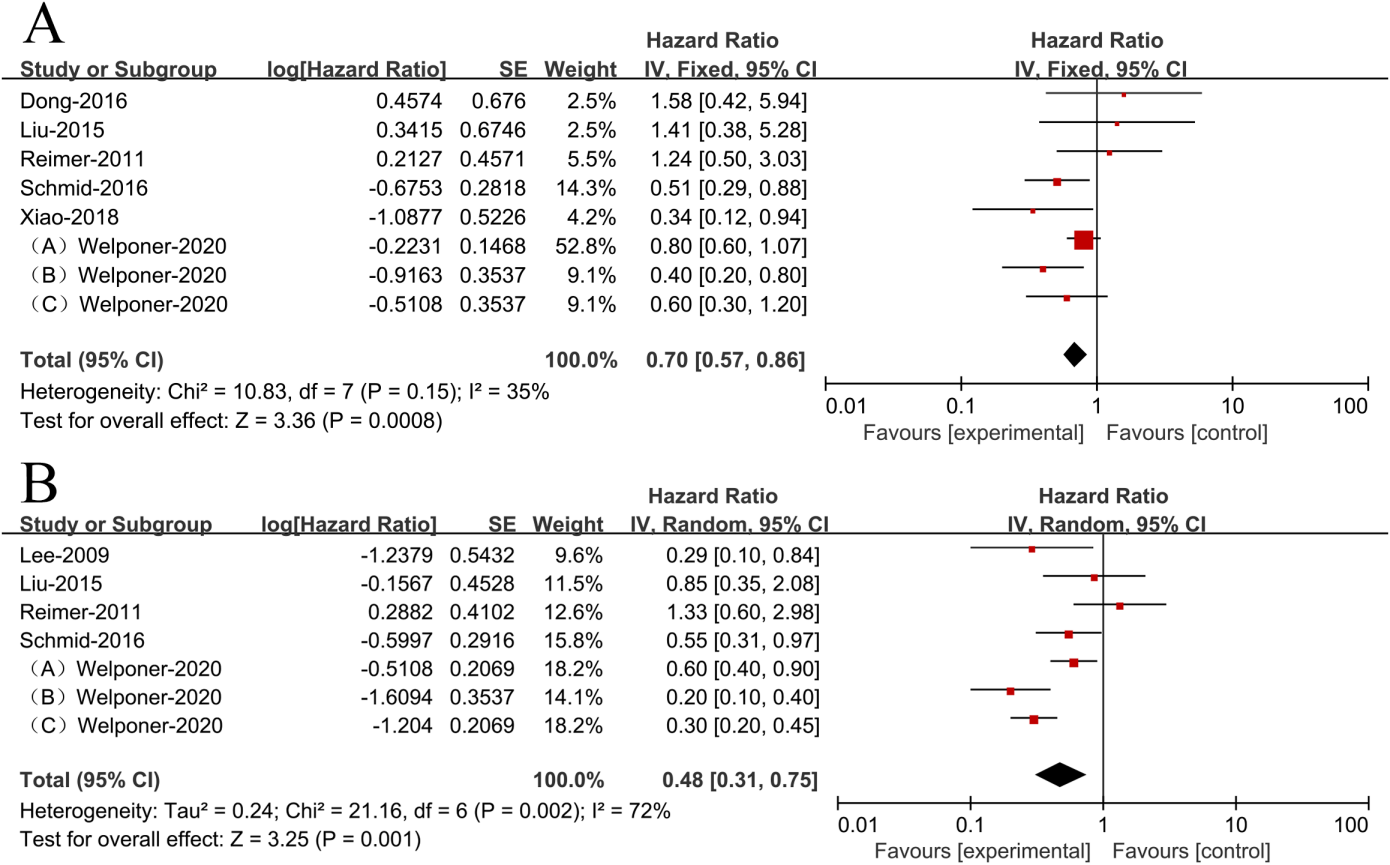
Patient survival plots for miR-34s expression in relation to overall survival **(A)** and progression-free survival **(B)**.

Since there was no heterogeneity in the included literatures (*I* ^2^ = 35%<50%, *p*>0.1), the analysis was conducted using a fixed-effects model. There was an association between higher miR-34s expression and improved OS (HR = 0.70,95% CI 0.57-0.86, **Figure 2A**) in women with ovarian cancer.

#### 3.3.2 PFS

Due to heterogeneity among the included literatures (*I* ^2^ = 72%>50%, *p <*0.1), analyzing the data was done using a random effects model. According to the results, ovarian cancer patients with higher miR-34s expression had significantly better PFS (HR = 0.48,95% CI 0.31-0.75, **Figure 2B**).

### 3.4 Subgroup analysis

**Figure 3:**
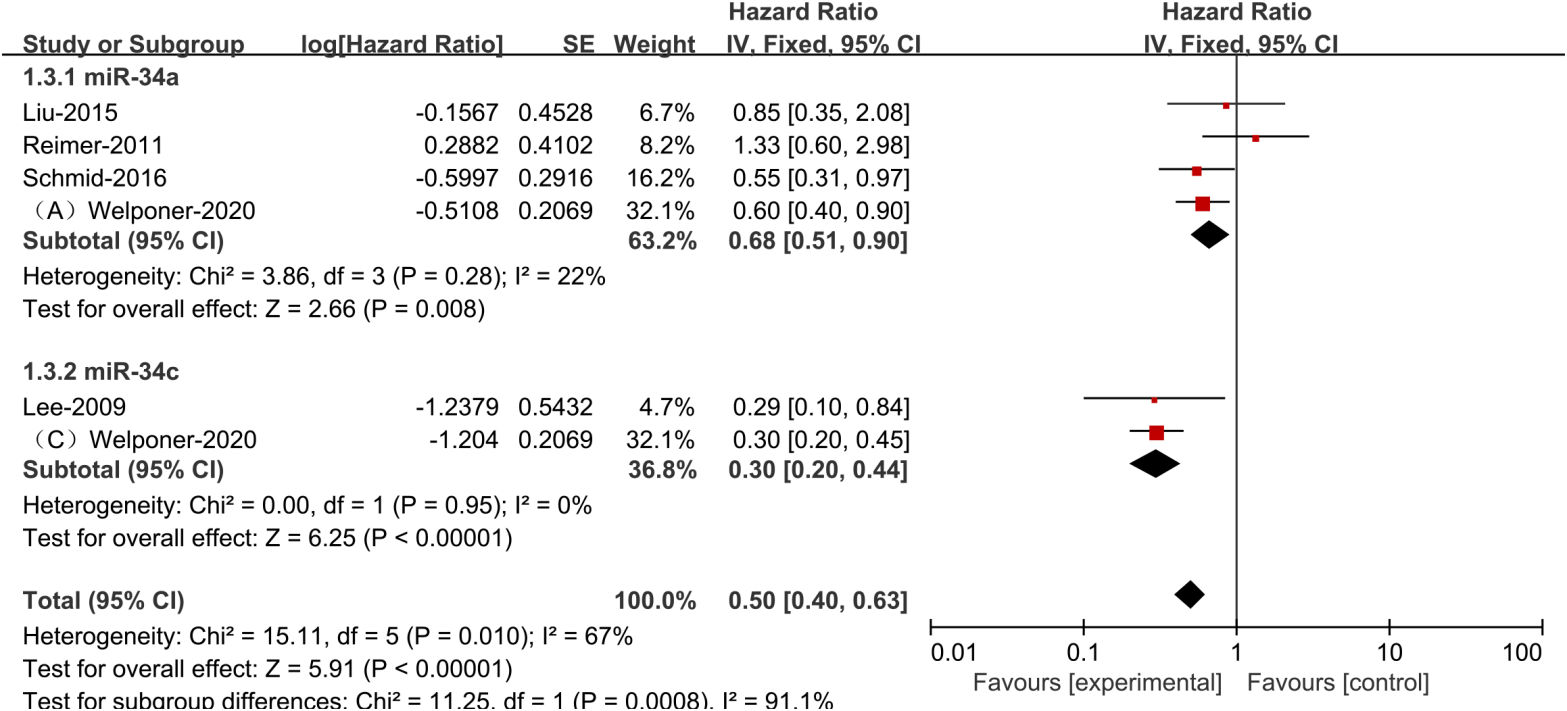
Plots showing specific miR-34s member expression and progression-free survival in ovarian cancer subgroups.

Since the outcome indicator PFS had significant heterogeneity, we conducted subgroup analysis to find the causes of heterogeneity. According to a stratified analysis by miR-34s member type, studies in the miR-34a and miR-34c groups showed no heterogeneity, while the heterogeneity between the three groups reached a high degree (*I* ^2^ = 67%, *p*<0.1, **Figure 3**), indicating that differences in the groups would greatly affect the results of meta-analysis.

### 3.5 Sensitivity analysis and publication bias

**Figure 4:**
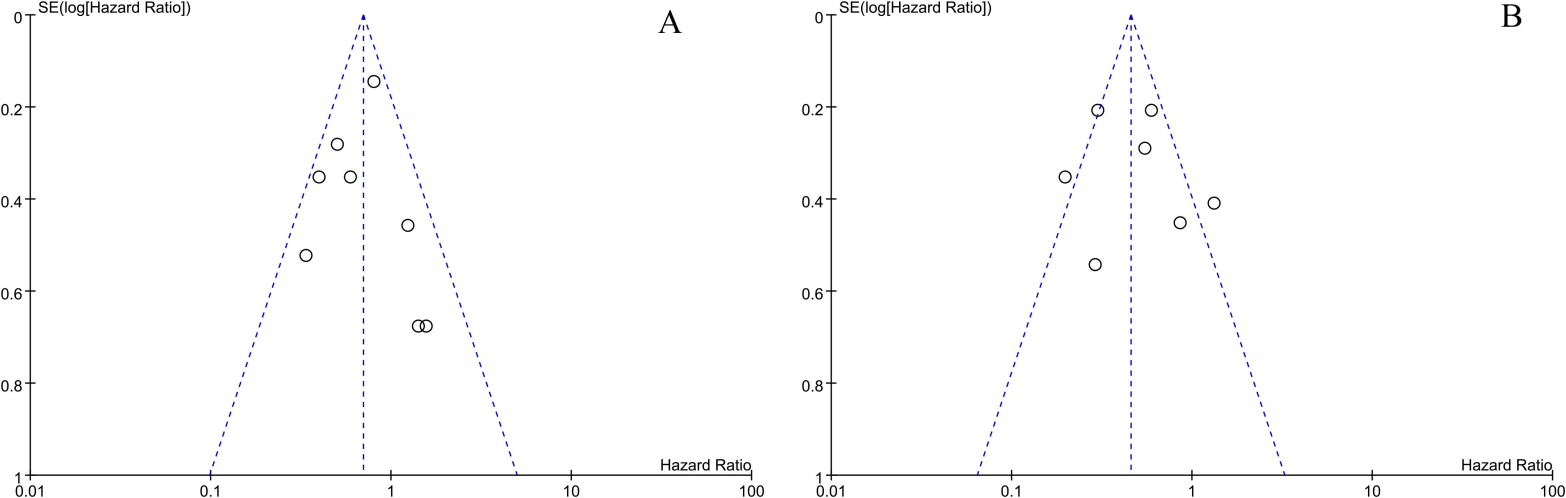
The overall survival and progression-free survival of miR-34s are depicted in a funnel plot **(A, B)**.

An analysis of OS and PFS sensitivity was conducted by eliminating individual studies, and it was found that no study had a significant impact on the overall results, and the results were relatively stable. According to both OS and PFS publication bias tests, it was found that the funnel plot was roughly symmetric and that there were no significant publication biases (**Figure 4**).

## 4 Discussion

Researchers have examined the relationship between miR-34s expression and survival in ovarian cancer patients. A total of 87 literatures were collected through six database and other ways, and 7 literatures were finally included. Firstly, a NOS quality assessment was conducted on the seven included studies, which were generally deemed to be of high quality. A pooled analysis of nine studies with 672 women, higher expression of miR-34s significantly increased OS and PFS for patients with ovarian cancer. Based on the significant heterogeneity of the outcome indicator PFS, stratified analysis by miR-34s member types identified miR-34a and miR-34c expression levels are higher as significantly associated with increased PFS. Lastly, sensitivity analysis found that no single study had a significant impact on overall results, and that the results were relatively stable and reliable. Using a publication bias test, the funnel plot was found to be roughly symmetrical, without evidence of publication bias.

The meta-analysis of research studies revealed that miR-34s expression was associated with a significant improvement in ovarian cancer patients’ OS and PFS. These findings align with the results reported by Schmid et al. (15), indicating that ovarian cancer cases with reduced miR-34a expression exhibited a decrease in PFS *(p* = 0.039) and OS (*p* = 0.016). Researchers have found that miR-34s expression levels are lower in cancerous tissues than non-tumorous tissues in ovarian cancer tissues (8). Ovarian cancer exhibiting elevated miR-34a and miR-34c levels may demonstrate reduced malignant potential, decreased invasiveness, and limited dissemination (8). Kwan et al. (17) observed there is a notable decrease in miR-34s expression in a number of malignant tumors, including breast cancer and colon cancer. They further demonstrated that this downregulation facilitated tumor suppression by modulating key molecular pathways involved in tumorigenesis. Inhibition of lung squamous cell carcinoma proliferation, migration, and invasion was associated with high miR-34s expression, according to Sun et al. (18) By targeting miR-34c, Wang et al. (19) demonstrated *in vivo* and *in vitro* that miR-34c inhibited malignant behaviors such as invasion, migration, proliferation, and epithelial-mesenchymal transformation of nasopharyngeal carcinoma. According to Elena et al. (20), it is possible that miR-34a can serve as an important indicator of recurrence in patients with non-small cell lung cancer. The diminished expression of miR-34s in osteosarcoma and hepatoblastoma has been correlated with an unfavorable prognosis (21, 22). Hence, it is believed that reduced miR-34s expression is a crucial factor in the initiation and progression of tumor growth.

We acknowledge that the heterogeneity for PFS in our study was high (I² = 72%), indicating significant differences among the included studies. To explore potential sources of this heterogeneity in more detail, we conducted a subgroup analysis to evaluate the impact of different RNA families on the results. Subsequently, we found that after performing the subgroup analysis, the I² value decreased to below 50%, suggesting that these subgroupings explain a substantial part of the heterogeneity. miR-34s represent the initial miRNA identified as being directly regulated by p53, with miR-34a demonstrating the most pronounced level of regulation by p53 (23). Furthermore, p53 and miR-34a expression is influenced by various indirect regulatory factors; for instance, miR-34a can enhance p53 activity by downregulating the expression of SIRT1, a NAD-dependent deacetylase involved in information regulation. Following the inhibition of p53 protein transcription, there is a subsequent down-regulation of p53 protein activity during the process of deacetylation (5, 24). In the context of epithelial-mesenchymal transition (EMT), miR-34 plays a crucial role by regulating the transcription factor Snail, which is pivotal in promoting EMT and invasion (16, 25). Additionally, miR-34a has been shown to negatively regulate E2F3a, a key transcription factor that promotes cell proliferation and facilitates the G1/S transition. In ovarian cancer cells, knockdown of miR-34a resulted in a significant increase in E2F3 expression, highlighting its role in cell cycle regulation (14). Moreover, miR-34a’s role extends to apoptosis, where its expression can influence apoptotic pathways, promoting cell death in cancer cells. This suggests that miR-34a not only acts as a tumor suppressor through cell cycle arrest and EMT regulation but also enhances apoptosis, thereby contributing to the suppression of malignant characteristics in ovarian cancer. For example, upregulation of miR-34a-5p has been shown to suppress the malignant characteristics of ovarian cancer cells by targeting TRIM44, impeding the advancement of the disease (26).Additionally, the miR-34 family members exhibit co-targeting of MET, a receptor protein tyrosine kinase, which influences the motility and invasion of epithelial ovarian cancer (27).Research has identified that MET is a target of miR-34c, enhancing the anti-tumor efficacy of cisplatin in ovarian cancer cells (28).In a study by Lu et al. (29), reverse transcriptional quantitative PCR demonstrated a significant reduction in exosome miR-34b expression in ovarian cancer cells, suggesting that exosome-derived miR-34b can diminish cell proliferation and hinder EMT in the SKOV3 ovarian cancer cell line. Based on these mechanistic studies, the findings collectively emphasize the importance of miR-34s in the cellular biology of ovarian cancer, particularly regarding their suppressive impact on proliferation, invasion, and promotion of apoptosis.

According to Kumar et al. (3), a MeDIP-NGS analysis revealed significant decreases in the relative expression levels of microRNA-34a in both tissue and serum samples of early epithelial ovarian cancer (*p*<0.0001). Furthermore, the functional analysis of microRNA-34a demonstrated that patients with stage III-IV and I-II epithelial ovarian cancer (EOC) had areas under curves of 92.0 (*p*<0.0001) and 82.7 *(p*<0.0001), respectively, suggesting that serum samples from these patients may be useful for monitoring cancer progression. In summary, miR-34s may serve as potential biomarkers in the diagnosis and prognosis of ovarian cancer. However, limitations such as varying sample sources, a paucity of literature, and inadequate sample sizes within studies may compromise the statistical robustness of miR-34s’ prognostic value. By controlling for certain objective conditions, it is feasible to accurately forecast the prognosis of miR-34s expression in ovarian cancer.

### 4.1 The advantages

To our knowledge, this is the first systematic review and meta-analysis of miR-34s’ prognostic value in ovarian cancer. From the methodological aspect, we conducted a two-person assessment, searched 6 databases, and further collected the literature comprehensively. We formulated inclusion and exclusion criteria according to PICOS principle, and the literature quality was relatively high.

### 4.2 The limitations

Due to the limited number of studies included in our analysis, consisting of three from China, three from Austria, and one from Canada, the data available for certain analyses and subgroup analyses was deemed insufficient. The findings of this study necessitate additional validation through large-scale, multicenter, multi-factorial, and high-quality clinical studies. Secondly, the potential for bias in the study may be influenced by variations in geographic regions and racial demographics. Thirdly, factors such as age, family history, and weight may also introduce bias.

## 5 Conclusions

Based on the results of this review, miR-34s could potentially serve as a prognostic indicator for an improved survival outcome for female ovarian cancer patients. However, due to the constraints of the present analysis, it is advisable to exercise caution when interpreting the conclusions. Additional clinical trials with rigorous methodology, a substantial sample size, and an extended follow-up period are necessary to further elucidate the prognostic significance of miR-34s expression in ovarian cancer.

## Data Availability

The original contributions presented in the study are included in the article/**Supplementary Material**. Further inquiries can be directed to the corresponding authors.
