# Supplementary material for: The prognostic value of miR-34 family in ovarian cancer: a systematic review and meta-analysis
Source: Front Oncol. 2025 Mar 17;15:1499163. doi: 10.3389/fonc.2025.1499163 (PMC11959189; doi:10.3389/fonc.2025.1499163)
Supplement: Supplementary file 1 [file DataSheet1.docx]

Supplementary Table 1. Cochrane Library, EMBASE, Web of Science, Wanfang, and CNKI.

| Database | Search strategy | |
| --- | --- | --- |
| Cochrane Library | #1 | 'ovarian neoplasms':ti,ab,kw OR 'carcinoma, ovarian epithelial':ti,ab,kw OR 'neoplasm, ovarian':ti,ab,kw OR 'ovarian neoplasm':ti,ab,kw OR 'ovary neoplasms':ti,ab,kw OR 'neoplasm, ovary':ti,ab,kw OR 'neoplasms, ovary':ti,ab,kw OR 'ovary neoplasm':ti,ab,kw OR 'neoplasms, ovarian':ti,ab,kw OR 'cancer, ovary':ti,ab,kw OR 'cancers, ovary':ti,ab,kw OR 'ovary cancers':ti,ab,kw OR 'ovary cancer':ti,ab,kw OR 'cancer, ovarian':ti,ab,kw OR 'cancers, ovarian':ti,ab,kw OR 'ovarian cancers':ti,ab,kw OR 'cancer of ovary':ti,ab,kw OR 'cancer of the ovary':ti,ab,kw OR 'epithelial carcinoma, ovarian':ti,ab,kw OR 'ovarian epithelial carcinomas':ti,ab,kw OR 'epithelial ovarian cancer':ti,ab,kw OR 'ovarian epithelial cancer':ti,ab,kw OR 'cancer, ovarian epithelial':ti,ab,kw OR 'epithelial cancer, ovarian':ti,ab,kw OR 'ovarian epithelial cancers':ti,ab,kw OR 'ovarian cancer, epithelial':ti,ab,kw OR 'cancer, epithelial ovarian':ti,ab,kw OR 'epithelial ovarian cancers':ti,ab,kw OR 'ovarian epithelial carcinoma':ti,ab,kw OR 'epithelial ovarian carcinoma':ti,ab,kw OR 'carcinoma, epithelial ovarian':ti,ab,kw OR 'epithelial ovarian carcinomas':ti,ab,kw OR 'ovarian carcinoma, epithelial':ti,ab,kw |
|  | #2 | 'MIRN34 microRNA, human':ti,ab,kw OR 'hsa-mir-34 microRNA':ti,ab,kw OR 'miR-34, human':ti,ab,kw OR 'MIRN34B microRNA, human':ti,ab,kw OR 'hsa-mir-34b microRNA':ti,ab,kw OR 'microRNA-34b, human':ti,ab,kw OR 'MIRN34C microRNA, human':ti,ab,kw OR 'hsa-mir-34c microRNA':ti,ab,kw OR 'miR-34c, human':ti,ab,kw OR 'microRNA-34c, human':ti,ab,kw OR 'miR-34b-3, human':ti,ab,kw OR 'miR-34c-5p, human':ti,ab,kw OR 'Pri-miR-34b-c, human':ti,ab,kw OR 'MIRN34A microRNA, human':ti,ab,kw OR 'miR-34a, human':ti,ab,kw OR 'microRNA-34a, human':ti,ab,kw OR 'hsa-mir-34a microRNA':ti,ab,kw OR 'microRNA 34a, human':ti,ab,kw |
|  | #3 | #1 AND #2 |
| EMBASE | #1 | 'ovarian neoplasms'/exp OR 'carcinoma, ovarian epithelial’:ab,ti OR “neoplasm, ovarian’:ab,ti OR “ovarian neoplasm’:ab,ti OR “ovary neoplasms’:ab,ti OR “neoplasm, ovary’:ab,ti OR “neoplasms, ovary’:ab,ti OR “ovary neoplasm’:ab,ti OR “neoplasms, ovarian’:ab,ti OR “cancer, ovary’:ab,ti OR “cancers, ovary’:ab,ti OR “ovary cancers’:ab,ti OR “ovary cancer’:ab,ti OR “cancer, ovarian’:ab,ti OR “cancers, ovarian’:ab,ti OR “ovarian cancers’:ab,ti OR “cancer of ovary’:ab,ti OR “cancer of the ovary’:ab,ti OR “epithelial carcinoma, ovarian’:ab,ti OR “ovarian epithelial carcinomas’:ab,ti OR “epithelial ovarian cancer’:ab,ti OR “ovarian epithelial cancer’:ab,ti OR “cancer, ovarian epithelial’:ab,ti OR “epithelial cancer, ovarian’:ab,ti OR “ovarian epithelial cancers’:ab,ti OR “ovarian cancer, epithelial’:ab,ti OR “cancer, epithelial ovarian’:ab,ti OR “epithelial ovarian cancers’:ab,ti OR “ovarian epithelial carcinoma’:ab,ti OR “epithelial ovarian carcinoma’:ab,ti OR “carcinoma, epithelial ovarian’:ab,ti OR “epithelial ovarian carcinomas’:ab,ti OR “ovarian carcinoma, epithelial’:ab,ti |
|  | #2 | 'MIRN34 microRNA, human'/exp OR ‘hsa-mir-34 microRNA’:ab,ti OR “miR-34, human’:ab,ti OR “MIRN34B microRNA, human’:ab,ti OR “hsa-mir-34b microRNA’:ab,ti OR “microRNA-34b, human’:ab,ti OR “MIRN34C microRNA, human’:ab,ti OR “hsa-mir-34c microRNA’:ab,ti OR “miR-34c, human’:ab,ti OR “microRNA-34c, human’:ab,ti OR “miR-34b-3, human’:ab,ti OR “miR-34c-5p, human’:ab,ti OR “Pri-miR-34b-c, human’:ab,ti OR “MIRN34A microRNA, human’:ab,ti OR “miR-34a, human’:ab,ti OR “microRNA-34a, human’:ab,ti OR “hsa-mir-34a microRNA’:ab,ti OR “microRNA 34a, human’:ab,ti |
|  | #3 | #1 AND #2 |
| Web of Science | #1 | (ALL=(Ovarian Neoplasms)) OR ALL=(Carcinoma, Ovarian Epithelial)) OR ALL=(Neoplasm, Ovarian)) OR ALL=(Ovarian Neoplasm)) OR ALL=(Ovary Neoplasms)) OR ALL=(Neoplasm, Ovary)) OR ALL=(Neoplasms, Ovary)) OR ALL=(Ovary Neoplasm)) OR ALL=(Neoplasms, Ovarian)) OR ALL=(Ovary Cancer)) OR ALL=(Cancer, Ovary)) OR ALL=(Cancers, Ovary)) OR ALL=(Ovary Cancers)) OR ALL=(Ovarian Cancer)) OR ALL=(Cancer, Ovarian)) OR ALL=(Cancers, Ovarian)) OR ALL=(Ovarian Cancers)) OR ALL=(Cancer of Ovary)) OR ALL=(Cancer of the Ovary)) OR ALL=(Epithelial Carcinoma, Ovarian)) OR ALL=(Ovarian Epithelial Carcinomas)) OR ALL=(Epithelial Ovarian Cancer)) OR ALL=(Ovarian Epithelial Cancer)) OR ALL=(Cancer, Ovarian Epithelial)) OR ALL=(Epithelial Cancer, Ovarian)) OR ALL=(Ovarian Epithelial Cancers)) OR ALL=(Ovarian Cancer, Epithelial)) OR ALL=(Cancer, Epithelial Ovarian)) OR ALL=(Epithelial Ovarian Cancers)) OR ALL=(Ovarian Epithelial Carcinoma)) OR ALL=(Epithelial Ovarian Carcinoma)) OR ALL=(Carcinoma, Epithelial Ovarian)) OR ALL=(Epithelial Ovarian Carcinomas)) OR ALL=(Ovarian Carcinoma, Epithelial) |
|  | #2 | **(ALL=(**MIRN34 microRNA, human**)) OR ALL= (**hsa-mir-34 microRNA**)) OR ALL=(**miR-34, human**)) OR ALL=(**MIRN34B microRNA, human**)) OR ALL=(**hsa-mir-34b microRNA**)) OR ALL=(**microRNA-34b, human**)) OR ALL=(**MIRN34C microRNA, human**)) OR ALL=(**hsa-mir-34c microRNA**)) OR ALL=(**miR-34c, human**)) OR ALL=(**microRNA-34c, human**)) OR ALL=(**miR-34b-3, human**)) OR ALL=(**miR-34c-5p, human**)) OR ALL=(**Pri-miR-34b-c, human**)) OR ALL=(**MIRN34A microRNA, human**)) OR ALL=(**miR-34a, human**)) OR ALL=(**microRNA-34a, human**)) OR ALL=(**hsa-mir-34a microRNA**)) OR ALL=(**microRNA 34a, human**)** |
|  | #3 | #1 AND #2 |
| Wanfang | 题名或关键词:(卵巢肿瘤 or 卵巢癌 or 上皮性卵巢癌组织 or 上皮性卵巢癌 or 上皮卵巢癌 or 卵巢上皮癌 or 卵巢恶性肿瘤 or 卵巢上皮性癌) and 题名或关键词:(miRNA-34 or miR-34 or microRNA-34 or MiR-34 or 微小RNA-34) | |
| CNKI | （主题：卵巢肿瘤（精确））OR（主题：卵巢癌（精确））OR（主题：上皮性卵巢癌组织（精确））OR（主题：上皮性卵巢癌（精确））OR（主题：上皮卵巢癌（精确））OR（主题：卵巢上皮癌（精确））OR（主题：卵巢恶性肿瘤（精确））OR（主题：卵巢上皮性癌（精确）） AND （（主题：miRNA-34（精确））OR（主题：miR-34（精确））OR（主题：microRNA-34（精确））OR（主题：MiR-34（精确））OR（主题：微小RNA-34（精确））） | |

Supplementary Table 2. NOS quality assessment results of the nonrandomized studies.

| Study, year | Study type | Representativeness  of the exposed cases | Selection of the nonexposed  casest | Ascertainment  of exposure | Outcome of interest was not present at  start of study | Comparability of cohorts on the basis of  the design or analysis | Assessment of outcome | Was follow-up long  enough for outcomes to occur | Adequacy of follow-up of cohorts | Total  score |
| --- | --- | --- | --- | --- | --- | --- | --- | --- | --- | --- |
| Lee [13] | Retro | 1 | 1 | 1 | 1 | 1 | 1 | 1 | 0 | 7 |
| Reimer [14] | Retro | 1 | 1 | 1 | 1 | 2 | 1 | 1 | 0 | 8 |
| Liu [9] | Retro | 1 | 1 | 1 | 1 | 2 | 1 | 1 | 0 | 8 |
| Schmid [15] | Retro | 1 | 1 | 1 | 1 | 2 | 1 | 1 | 0 | 8 |
| Dong [16] | Retro | 1 | 1 | 1 | 1 | 0 | 1 | 1 | 0 | 6 |
| Xiao [10] | Pro | 1 | 1 | 1 | 1 | 2 | 1 | 1 | 0 | 8 |
| Welponer [8] | Retro | 1 | 1 | 1 | 1 | 2 | 1 | 1 | 0 | 8 |
| Welponer [8] | Retro | 1 | 1 | 1 | 1 | 2 | 1 | 1 | 0 | 8 |
| Welponer [8] | Retro | 1 | 1 | 1 | 1 | 2 | 1 | 1 | 0 | 8 |
